# Can the Use of Health Insurance Claim Data Benefit the Risk-Based Supervision of General Practitioner Practices? An Exploratory Study in the Netherlands

**DOI:** 10.34172/ijhpm.2020.242

**Published:** 2020-12-19

**Authors:** Rudolf Bertijn Kool, Reinier Peter Akkermans, Ine Borghans, Corline Brouwers, Sander Ranke

**Affiliations:** ^1^Radboud University Medical Center, Radboud Institute for Health Sciences, IQ Healthcare, Nijmegen, The Netherlands.; ^2^Radboud University Medical Centre, Department of Primary and Community Care, Nijmegen, The Netherlands.; ^3^Dutch Health and Youth Care Inspectorate, Utrecht, The Netherlands.; ^4^Department of Public and Occupational Health, Amsterdam Public Health Research Institute Amsterdam UMC, Vrije Universiteit Amsterdam, Amsterdam, The Netherlands.

**Keywords:** Supervision of Healthcare, Risk Assessment, The Netherlands, General Practitioner

## Abstract

**Background:** The Dutch Health and Youth Care Inspectorate has organized a study investigating whether there are benefits to using claim data in the risk-based supervision of general practitioner (GP) practices.

**Methods:** We identified and selected signals of risks based on interviews with experts. Next, we selected 3 indicators that could be measured in the claim database. These were: the expected and actual costs of the GP practice; the percentage of reserve antibiotics prescribed; and the percentage of patients undergoing an emergency admission during the weekend. We corrected the scores of the GP practices based on their casemix and identified practices with the most unfavorable scores, ‘red flags,’ in 2015, or the trend between 2013-2015. Finally, we analysed the data of GP practices already identified as delivering substandard care by the Health and Youth Care Inspectorate and calculated the sensitivity and specificity of using the indicators to identify poor performing GP practices.

**Results:** By combining the 3 indicators, we identified 1 GP practice with 3 red flags and 24 GP practices with 2 red flags. The a priori chance of identifying a GP practice that shows substandard care is 0.3%. Using the indicators, this improved to 1.0%. The sensitivity was 26.7%, the specificity was 92.8%.

**Conclusion:** The Dutch Health and Youth Care Inspectorate might use claim data to calculate indicators on costs, the prescribing of reserve antibiotics and emergency admissions during the weekend, when setting priorities for its visits to GP practices. Visiting more GP practices by the Health and Youth Care Inspectorate, and identifying substandard care, is necessary to validate the use of these indicators.

## Background

**Key Messages**

** Implications for policy makers**

- Healthcare inspectorates might use health insurance claim data to prioritise visits to healthcare providers when the number of providers is too large to visit them all.
- Using health insurance claim data for assessing performance problems is an interesting option without administrative burden for healthcare professionals.
- Costs, the prescribing of reserve antibiotics and emergency admissions during the weekend, can be used to set priorities for general practitioner (GP) supervision.

** Implications for the public**

The supervision of general practitioners (GPs) by the Healthcare Inspectorate is challenging because of the major amount of healthcare providers. In this study, we explored whether health insurance claims could help the Inspectorate in identifying GPs with a higher risk of substandard care. We selected 3 indicators that could be measured in the claim database. These were: the expected and actual costs of the practice; the percentage of reserve antibiotics prescribed; and the percentage of patients undergoing an emergency admission during the weekend. Our results show that these data may be used to prioritise visits by the Inspectorate. These visits are necessary to validate supervision based on assessment of risks using health insurance claim data.

In many countries, healthcare inspectorates struggle with the organization of supervision based upon risk.^1^ Such risk-based supervision aims to predict efficiently, accurately, and timely, risks to the quality and safety of healthcare.^2^ It facilitates effective supervision by prioritizing efforts to control the quality of healthcare and allows for regulatory activity to be directed at the most high-risk services.^3^ By targeting their inspections, inspectorates can diminish administrative burdens and optimize proportional regulation.^4^ A potential disadvantage of risk-based supervision is a failure to outline a realistic image of medical risk to patients.^5^ Because risk-based supervision is deemed to be efficient, it might be especially suitable for the supervision in healthcare sectors with a large number of providers, such as general practitioners (GPs), dentists and midwives. Resources that can be used for risk-based supervision of these healthcare professionals are often scarce. In England, the Care Quality Commission (CQC) has made several attempts to implement a risk-based approach. It used data on quality to feed statistical surveillance tools in order to predict risks and to actually regulate the 30 000 health and social care providers.^6^ The UK experiences showed the challenge of prioritising inspections based on statistical surveillance: Griffiths et al concluded that such intelligent monitoring was not yet suitable for this purpose and might result in more harm if poor-quality care was undetected for long periods in healthcare providers that the CQC mistakenly believes to be low risk.^6^

 There are several conditions for risk-based supervision which makes implementation in healthcare challenging. Firstly, risk-based supervision implies being able to identify the risk of substandard care and having a consensus on that risk.^1^ In healthcare, this is an important challenge because of the intense debate on what quality of care is exactly. Secondly, risk-based supervision needs valid and easily accessible data that allows quantification of the risk.^7^ Thirdly, risk-based supervision demands an acceptance by society of the ongoing risks to patients from providers who will not be subject to the supervision.^8^

 The Dutch Health and Youth Care Inspectorate is also looking for ways of implementing risk-based supervision. It has already introduced an extensive monitoring system for hospitals and other care providers. It uses information collected by the Inspectorate, as well as information from external databases, such as patient rating sites.^9,10^ However, the data available for risk-based supervision of GP practices is limited and so the data collected from health insurance claims could be an interesting source for such supervision. These data score relatively highly on reliability and completeness because healthcare providers are financially dependent on how accurate and complete these data are. In the Netherlands, there is a national database of health insurance claims. It contains reimbursement data on all medical diagnoses and treatment procedures paid for by Dutch health insurance companies, including those by individual healthcare professionals. Healthcare insurance is compulsory so almost all Dutch inhabitants have a private healthcare insurance offered by 1 of the 10 healthcare insurance companies.

 Since 2017, the Dutch Health and Youth Care Inspectorate has been allowed to use this national database of insurance claims in order to improve the supervision of healthcare. Therefore, it organized a study to investigate whether there are benefits to using claim data in risk-based supervision, and selected GP care as a test case. Besides delivering basic primary care, the GP should identify and refer patients with potentially serious risks because of the important gatekeepers’ role of GPs in Dutch healthcare. The aim of this study is to explore whether data from this health insurance claim database could identify GP practices with a higher risk of substandard care, and whether this information could serve as a base for prioritizing visits by the Inspectorate to GP practices.

## Methods

 Firstly, in order to identify which risks might be measurable using claim data, we interviewed the 2 senior healthcare inspectors for GP care (n = 2), representatives of the 2 Dutch GP professional bodies (n = 4), and researchers active in the field of primary care (n = 4). We chose the 4 experts based on their previous publications on this topic. Secondly, in order to select risks for substandard GP care that are measurable in the claim database, we interviewed data analysts involved with the claim database (n = 3) and then selected 3 indicators based on this interview. Thirdly, using the data from the claim database, we analyzed the 3 indicators we had selected.

###  Identifying and Selecting Signals of Risks

 In order to identify the risks in GP care that would be measurable with claim data, we made an inventory of topics based on the existing literature. One researcher (SR) made the topic list and the other 4 researchers (RBK, RPA, IB and CB) commented on this. Based on the topic list (see Supplementary file 1), we developed a semi-structured interview guideline. Four researchers (RBK, RA, CB and SR) conducted the face to face interviews in pairs. All interviewers were trained and experienced in interviewing healthcare professionals and had no connection with the interviewees. The interviews were recorded and one of the researchers present at the interview made a summary. This was, in turn, checked by the other researcher who was present at the interview. We sent the summary to the interviewee to confirm a correct interpretation of the answers that were given. Next, we identified signals that are a possible risk to good GP practice which were mentioned 3 times or more by the interviewees. Lastly, we discussed these signals with the 3 data analysts, who all work on the claim database every day, in order to assess whether these signals were measurable using the existing database. This could be either in a single variable, a combination of variables, or a proxy variable. We checked with the data analysts both the availability of the necessary data in the claim database and whether there was a need for corrections due to the casemix.

 From the interviews with the GP inspectors, representatives of the GP professional bodies, and the experts, we identified the following 4 signals which could provide an indication for substandard GP care;

1. GP practices that generate extreme (high and low) costs in their own practice or elsewhere, might indicate a low adherence to guidelines.
2. Extremes in prescribing behavior of GPs. In particular, a relatively high number of prescriptions for pain medication, psychopharmaceuticals, antibiotics and morphine might be a signal for low adherence to guidelines. GPs often prescribe these medications and so there are clear Dutch GP guidelines on when and how much to prescribe. The prescribing of antibiotics might, in particular, give the Inspectorate valuable information about the risk of poor GP care because of the considerable attention given in the Netherlands to restricting the use of antibiotics. Of this category, the so-called reserve antibiotics, might be the most informative. Dutch GP guidelines advise prescribing antibiotics with a small spectrum: the treatment should be focused on the specific bacteria. This will cause less resistance to antibiotics. Antibiotics with a broader spectrum such as amoxicillin with clavulanic acid or fluoroquinolones are meant as reserve antibiotics and should only be used when the first choice shows no effect. Reserve antibiotics are also prescribed for infections difficult to treat such as amoxicillin with clavulanic acid for an aspiration pneumonia and ciprofloxacin for urinary tract infections with tissue invasion.^11^
3. An extreme number of patients belonging to a GP practice who require an emergency admission to a hospital during the weekend might indicate an insufficient organization of the care for chronic patients within that GP practice. Research has shown that the way GP practices are organized influences the use of emergency services during out of office hours.^12^
4. An extreme workload experienced by the employees of the GP practice might be a risk factor and a signal for the Inspectorate to visit that practice.

We concluded, based on the interviews with the database experts, that the first 3 signals mentioned above were measurable since the necessary data – including data for casemix correction – were available in the database. The fourth signal could not be measured because the database does not contain data on the number of full-time equivalents of GPs, assistants and practice nurses.

 Therefore, we formulated the following 3 indicators for which we requested the health insurance data:

1. The expected and actual total costs per patient for, 2013, 2014 and 2015 and their 4 sub-categories: GP practice costs, costs of diagnostics of the GP practice, hospital costs, and pharmaceutical costs.
2. The percentage of prescribed reserve antibiotics divided by the total volume of prescribed antibiotics.
3. The percentage of patients of a GP practice for whom a claim code was opened for a hospital admission during the weekend (Friday 6 PM until Monday 9 AM).

###  Analysis of Claim Data

 We analyzed the data regarding the 3 indicators selected from the claim database for the 3 most recent complete years (2013, 2014 and 2015). The primary goal was to focus on identifying the practices with the most unfavorable scores on these indicators, thereby maximizing the yield for the Inspectorate. Therefore, we targeted the extremes of all 3 indicators. We received permission of VEKTIS, the supervising body of the claim database, to use the data. The data we analyzed were made anonymous.

 For the first indicator, the expected and actual costs were per practice for every registered patient. There were 5 categories of costs: total costs, costs related to GP care, costs related to hospital care, costs related to pharmaceutical care and costs related to diagnostics performed in primary care. The costs were corrected for age, sex, socioeconomic status (based on postal code and divided into 3 categories high, middle and low), number of prescribed medicines, and the number of patients with 3 medical conditions – chronic obstructive pulmonary disease, cardiovascular diseases, and diabetes mellitus. For constructing the cost-indicator, first, the difference between the expected and actual costs per patient were calculated for the most recent year (2015). Second, the difference between the *actual costs per patient* were calculated between 2013-2015. Because the aim of our research was to select healthcare providers with extreme high or low costs, we chose the difference between expected and observed costs instead of the ratio. Based on these results the GP practices were divided into 3 groups; a group of practices with lower costs than expected (1st percentile), a group of practices with higher costs than expected (99th percentile), and a group of practices with scores in the middle range. Since both extreme low costs as well as extreme high costs were considered to be of interest we calculated the number of categories on which each practice scored very low or very high. If a practice scored low or high on at least 2 different categories the practice was given an orange flag, in case the practice scored high or low on 3 different categories the practice was given a red flag.

 The second indicator (percentage prescribed reserve antibiotics), and the third indicator (percentage emergency admissions during the weekend) were corrected for casemix using the following data from all GP practices: the year; the total number of patients; their socioeconomic status (low, middle, high); their age categories (0-4, 5-17, 18-44, 45-64, 65-74, 75+ years); their sex; and the number of patients with chronic obstructive pulmonary disease, cardiovascular diseases and diabetes mellitus that may vary between the GP practices and have a major influence on care consumption. We used the data from these variables for 2013, 2014 and 2015, in a logistic regression model to calculate a corrected score for each GP practice. For every GP practice, the percentages of the second and third indicator were calculated as the total number of patients with the relevant aspect (event), divided by the total number of patients in the GP practice (trial). The event and trial were used as variables which describe the dependent variable (event-and-trial syntax) in the logistic regression analysis, and the abovementioned correction variables as independent variables (procedure generalized linear models in SPSS 21). The expected number of patients was calculated by adding up the patients’ individual probabilities in a GP practice. The corrected score for a GP practice was calculated by dividing the total number of patients with the relevant aspect by the expected number of patients with the relevant aspect (based on the correction from the logistic regression analysis) and multiplying this by the national score. We calculated the national score by dividing the sum of all nominators by the sum of all denominators.

 For the second indicator, the percentage of reserve antibiotics, and the third indicator, the percentage emergency admissions during the weekend, only the highest extreme was considered to be of interest. We, therefore, used the 98th percentile for these indicators. The practices were ranked in each of the 3 indicators for the most recent year, 2015. We also ranked the practices based on the trend between 2013 and 2015. This was because sudden changes in practice scores might also represent a risk and could be a signal for the Inspectorate to intervene. A practice was given a red flag if its score in 2015, or the trend between 2013 and 2015, would belong to the 1st, 98th or 99th percentile, depending on the indicator. If not, the practice was given a green flag. For one of the indicators, the costs, which had 5 sub-categories, we gave a GP practice a red flag when 3 or more cost sub-categories recorded an extreme score in the 1st or 99th percentile. At the end, we counted the red flags for each practice. Our assumption was that practices with an extreme score might be practices that show signs of poor quality of care, the practices that are interesting for the Inspectorate.

 Our aim was to characterize the practices which had an extreme score. We achieved this by comparing the practices with 2 or 3 red flags with the other practices with regard to the following characteristics: the total number of patients; their socioeconomic status (low, middle, high); their age categories (0-4 years, 5-17, 18-44, 45-64, 65-74, 75+); their sex; and the number of patients with the medical conditions - chronic obstructive pulmonary disease, cardiovascular diseases and diabetes mellitus. Because of the multiple testing of categories of Socioeconomic status and age a Bonferroni correction was be applied.

 Finally, we validated the indicators using the scores of GP practices that were already under investigation by the Health and Youth Care Inspectorate because they were a risk for patient safety. The Inspectorate indicated us anonymously these practices with problems and we added the indicator scores of these practices. We then identified whether there were statistically significant more extreme scores in this subgroup of practices with problems. We then calculated using Bayes’ theorem (P(A|B) = P(B|A)*P(A)/P(B)), the chances of identifying a GP practice that shows substandard care with or without the indicators. Finally, we calculated the sensitivity, the specificity, the positive predictive value and the negative predictive value.

## Results

Table 1 shows the difference between the expected and actual costs per patient that were calculated for the most recent year (2015). The mean actual total costs per patient in 2015 was 1478 euros with a variation of 84 to 8711 euros. The mean expected costs per patient in 2015 was 1505 euros with a variation of 444 to 12689 euros. The mean cost difference for the total costs is negative (-28 euros). The cost difference for the categories GP practice costs, GP diagnostics costs and pharmaceutical costs were (slightly) positive. In Table 2 the mean cost difference between the actual costs over the years was negative for the total costs, the GP diagnostic costs and the hospital costs meaning that the actual costs per patient in 2015 were slightly lower compared to 2013. Notable are the extremes in the minimum and maximum costs (total costs and hospital costs) compared to the 1st and 99th percentile.

**Table 1:** Difference Between Actual and Expected Costs (in euros) and the 5 Categories of the GP Practices

**N 2015 = 4933**	**Total Costs**	**GP Diagnostics**	**Hospital Costs**	**GP Practice Costs**	**Pharmaceutical Costs**
Mean	-27.67	0.84	-18.70	1.89	0.73
Median	-29.57	-0.42	-18.25	-0.02	-0.81
Minimum	-3977.78	-50.87	-1458.40	-80.94	-2109.84
1st percentile	-262.26	-15.87	-185.66	-42.12	-33.96
99th percentile	252.39	32.19	151.40	53.17	53.25
Maximum	1476.04	125.44	512.29	608.33	560.74

Abbreviation: GP, general practitioner.

**Table 2:** Difference Between the Actual Costs Per Patient Between 2013-2015 for the 5 Categories of the GP Practices

**N 2015 = 4933**	**Total Costs**	**GP Diagnostics**	**Hospital Costs**	**GP Practice Costs**	**Pharmaceutical Costs**
Mean	-14.40	-4.50	-58.71	2.15	9.15
Median	-14.93	-2.93	-56.86	0.70	8.28
Minimum	-3312.97	-54.03	-1272.75	-123.43	-186.19
1st percentile	-212.23	-32.14	-206.67	-40.29	-16.15
99th percentile	187.88	13.15	79.65	55.53	40.85
Maximum	3098.61	112.44	879.60	612.14	128.51

Abbreviation: GP. general practitioner.

 Based on Table 1 there were 0.2% (n = 11) practices that scored very low on at least 3 categories and 0.4% (n = 18) practices that scored very high on at least 3 categories. Five other practices showed a combination of very low and very high scores on at least 3 categories. In total n = 34 practices had extreme scores.

 Based on Table 2 there were 0,3% (n-14) practices that scored very low on at least 3 categories and 0.2% (n = 12) that scored very high on at least 3 categories. Again, 5 practices showed a combination of very low and very highs scores on at least 3 categories. In total n = 31 practices had extreme scores. Since there was a partial overlap (n = 8) between the practices with extreme scores (Tables 1 and 2) n = 57 practices were left with a red flag on cost-indicator.

Tables 3 and 4 show the percentage of reserve antibiotics and the change in percentage of reserve-antibiotics between 2013-2015. On average, around 16% of the antibiotic prescriptions given by a GP were for reserve antibiotics. The mean change in the percentage of reserve antibiotics between 2013 and 2015 is 3%.

**Table 3:** The Percentage of Reserve-Antibiotics Prescribed (Based on Total Volume of Reserve-Antibiotics Divided by Total Volume of Prescribed Antibiotics)

	**2013** **(N = 4836)**	**2014** **(N = 4858)**	**2015** **(N = 4921)**
Mean (SD)	16.2% (5.9%)	16.3% (6.3%)	16.2% (5.7%)
Median	15.2%	15.3%	15.3%
Minimum	0.0%	0.0%	0.0%
98th Percentile	31.5%	31.8%	31.4%
Maximum	100.0%	100.0%	100.0%

Abbreviation: SD, standard deviation.

**Table 4:** Percentage Change in Reserve Antibiotics Between 2013-2015

	**% Change Reserve Antibiotics ** **(N = 4402)**
Mean (SD)	103.4 (25.6)
Median	100.9
Minimum	0.0
95th Percentile	147.1
98th Percentile	165.1
Maximum	269.3

Abbreviation: SD, standard deviation.

 We identified n = 98 practices which had scores in the 98th percentile in 2015. In addition, we found n = 88 practices which scored extreme on the trend in prescription. In total, n = 183 unique practices received a red flag based on the second indicator.

Table 5 shows the percentage of patients who underwent an emergency admission during the weekend; and the change in the percentage of patients who underwent an emergency admission during the weekend between 2013 and 2015. On average, 2.9% of the patients had an emergency admission during the weekend. The change in the percentage of emergency admissions between 2013 and 2015 is 150%, while the median is at 101%. This is due to practices with extremely high scores.

**Table 5:** The Percentage of Patients Who Underwent an Emergency Admission During the Weekend

	**2013** **(N = 4837)**	**2014** **(N = 4889)**	**2015** **(N = 4361)**
Mean (SD)	2.9 (1.05)	2.9 (1.4)	150.1 (712.4)
Median	2.8	2.9	100.8
Minimum	0.0	0.0	0.0
98th Percentile	4.4	4.2	141.1
Maximum	49.7	89.9	23 583.5

Abbreviation: SD, standard deviation.

 There were n = 97 practices with extreme scores on the percentage of weekend emergency admissions (98th percentile) in 2015 and n = 87 practices with extreme scores on the percentage change between 2013-2015 on the number of weekend admissions. A total of n = 179 of unique practices received a red flag on the third indicator.

 Combining the 3 indicators, we identified 1 GP practice with 3 red flags and 24 GP practices with 2 red flags. Table 6 compares those GP practices with 2 or 3 red flags with the other GP practices with regard to the casemix variables. There are differences between the GP practices with 2 or 3 red flags and the other practices regarding the number of GP’s, sex, SES and age.

**Table 6:** Comparison of the GP Practices With 2 or 3 Red Flags With the Other GP Practices With Regard to the Casemix Variables

	**Two/Three Red Flags Practices** ^a^	**Other Practices** ^b^	***P *** **Value**
Mean number of patients (SD)	1573 (2035)	3010 (1805)	.46
Mean number of GPs (SD)	1.40 (0.82)	1.59 (1.24)	<.001
Socioeconomic status			
% Low (SD)	38 (24)	30 (14)	.007
% Middle (SD)	39 (16)	38 (8)	.53
% High (SD)	26 (17)	32 (14)	.07
% Female	53 (9)	51 (2)	<.001
Age			
% Age 0-4	8 (10)	5 (2)	<.001
% Age 5-17	16 (5)	15 (3)	.20
% Age 18-44	36 (12)	33 (6)	.004
% Age 45-64	26 (7)	29 (4)	.006
% Age 65-74	10 (6)	11 (3)	.16
% Age >75	11 (18)	8 (3)	<.001
% COPD	2 (2)	3 (1)	.07
% CVRM	24 (15)	22 (5)	.05
% Diabetes	7 (4)	6 (2)	.001

Abbreviations: SD, standard deviation; GP, general practitioner; COPD, Chronic obstructive pulmonary disease; CVRM, cardiovascular risk management. ^a^Number of GP practices used for analyses ranged between n = 19-25. ^b^Number of GP practices used for analyses ranged between n = 4881-5359.

Table 7 shows the indicator scores of the GP practices that are already under surveillance by the Health and Youth Care Inspectorate because of a notification of substandard care. Four GP practices scored statistically significant differently.

**Table 7:** Indicator Scores of the 15 GP Practices Delivering Substandard Care

**No.**	**GPs (n)**	**Patients (n)**	**Reserve Antibiotics 2015 as Percentage of Total Antibiotics (%)**	**Reserve Antibiotics 2013-2015 change (%)**	**Emergency Admissions 2015 (%) **	**Emergency Admissions 2013-2015 change (%)**	**Total Costs 2015 (€)** ^a^	**Total Costs 2015-2013 (€)**	**Red Flag**
1	2	3256	143/644 (22.2)	137.5%	3.7%	105.3%	49.52	107.66	0
2	1	1821	105/608 (12.1)	114.9%	3.9%	86.4%	182.17	-21.97	0
3	2	2343	140/542 (25.8)	187.8%*	2.5%	117.0%	-114.75	-49.40	1
4	3	1030	2/5 (40.0)*	-	-	-	-83.27	-	1
5	1	2044	61/432 (14.1)	86.3%	4.4%*	117.1%*	106.02	20.47	1
6	2	2729	95/828 (11.5)	100.2%	3.4%	117.9%	-78.24	-90.06	0
7	1	2523	272/908 (30.4)	96.1%	2.1%	80.0%	12.33	40.91	0
8	1	1555	101/593(17.0)	108.5%	2.8%	89.2%	-1.97	-11.79	0
9	1	2189	72/824 (8.7)	53.2%	3.8%	106.4%	331.78*	180.04	1
10	2	3532	95/638 (14.9)	86.7%	2.3%	128.6%	3.51	63.55	0
11	2	4513	14/46 (30.4)	97.1%	-	-	-156.74	21.72	0
12	1	3451	63/875 (7.2)	89.4%	3.1%	110.3%	-16.41	-72.86	0
13**	-	-	-	-	-	-		-	0
14	4	8729	327/2384 (13.7)	91.3%	3.0%	103.5%	71.40	-27.03	0
15	2	3498	117/758 (15.4)	124.0%	3.1%	100.8%	-25.79	-86.59	0

Abbreviation: GP, general practitioner. * Significant (red flag; 98^th^ percentile on antibiotics and emergency admissions or at least 3 categories of extreme low or high costs. ** No data available. ^a^Only the total costs are depicted in the table.

Table 8 shows the data for the calculation of the sensitivity and specificity of the combination of indicators.

 We can calculate using Bayes’ theorem (P(A|B) = P(B|A)*P(A)/P(B)), the chances of identifying a GP practice that shows substandard care with or without the indicators. The a priori chance of identifying a GP practice that shows substandard care is 0.3% (15/5384). Using the indicators, this improves to 1.0% (4/15*15/ 5384)/(393/5384). The sensitivity is 26.7% (4/15), the specificity is 92.8% (4980/5369). The positive predictive value is 1.0 % (4/393) and the negative predictive value is 99.8 % (4980/4991).

**Table 8:** Sensitivity and Specificity of Using the 3 Indicators to Detect Substandard Care

	** GP Practices Without Red Flag (n)**	**GP Practices With Red Flag (n)**	**Total**
No notification of substandard care	4980	389	5369
Notification of substandard care	11	4	15
Total	4991	393	5384

Abbreviation: GP, general practitioner.

## Discussion

 In this study, we explored whether data from a health insurance claim database could be used in risk-based supervision to select which GP practices the Health and Youth Care Inspectorate might visit in order to increase the chance of finding substandard care. From the interviews, 4 themes emerged: GP practices that generate extreme costs in their own practice or elsewhere; extremes in prescribing behavior of GPs – in particular pain medication, psychopharmaceuticals, antibiotics and morphine; an extremely large number of patients from a GP practice who undergo an emergency admission to a hospital during the weekend; and finally, an extremely high workload experienced by the employees of the GP practice. The first 3 could be measured in the claim database by calculating an indicator including casemix correction.

 The indicators identified 25 GP practices with 2 or 3 red flags that might be visited by the Inspectorate. Applying the red flags to the GP practices that were already identified as delivering substandard care, showed that by using the 3 indicators the a priori chance of identifying substandard care increased by more than 3 times. However, it resulted in a low sensitivity. This is caused by the small number of GP practices with substandard care in relation to the total number of GP practices in the Netherlands. It seems necessary to visit several practices with 2 or 3 red flags and identify more practices with substandard care in order to enhance the sensitivity of the 3 indicators. Therefore, the finding that by using the 3 indicators, the a priori chance increased more than 3 times should be interpreted with caution.

 We used several confounders for our casemix that are known to be of influence on the outcomes. The casemix correction might be improved because other unknown factors related to the patient might play a role in assessing the outcome. Regional factors could influence the results. In the Netherlands, there are, for example, regional differences in prescribing antibiotics.^13^

 It is significant that many of the 25 GP practices with red flags had a small number of patients. There are several explanations for this finding. Firstly, some GPs are working for 2 or more practices. Their patients cannot be allocated to one practice and therefore they are excluded from the analyses. If a GP practice works with several GPs working for more practices, this might result in a relatively small number of patients in the claim database. Secondly, the GP practice may be based in Germany or Belgium and, therefore, has only a few Dutch patients. The other, foreign, patients will not be included in the Dutch claim database.

###  Comparison With Existing Literature

 There is hardly any literature known about trying to prioritize supervision by healthcare inspectorates. Griffiths et al^6^ showed that the statistical surveillance tool that the CQC was using in the United Kingdom, was not able to predict the outcome of National Health Service hospital trust inspections. This disappointing result shows the difficulty of predicting risks. The Dutch healthcare Inspectorate recently concluded that text mining of a complaint database might be an option for predicting risks and prioritizing visits.^14^

###  Strengths and Weaknesses

 An important strength of our research is that the database consists of data from all practicing GPs in the Netherlands. However, the data could only be interpreted on a practice level, not on an individual GP level. Many practices with 2 GPs or more register their data by claiming all consultations by only 1 GP. This makes reporting on an individual level unreliable. Another weakness is the number of missing values. For most categories, the number of missing values is 8%-9% and for the trend data it is almost double that figure. Also, using casemix correction for performance measurement has serious challenges because of the limitations of databases. There may be factors of which we did not have any data and that influenced the outcomes on the different indicators. Quality of primary care is not only influenced by the GP care itself but also by the organization of care within the region and various patient factors (for example the collaboration between care providers, lack of social support, and the quality of information/communication) which is not available in the database.

 The ‘flagging approach’ has some limitations, eg, the choice of the thresholds used is often subjective and arbitrary.^15^ An alternative way of identifying practices for inspection might be using the Z-score of all the different performance measures instead of using 98%/99% cut-off rates.^16^ However, for this method we need enough practices with substandard care. When the Inspectorate has gathered more data of practices with substandard care, it would be interesting to use this Z-score to analyse whether the same practices would be identified as potential poor performers. Finally, no recent data was available, due to a delay in the registration. The longer the delay, the higher the probability to miss the identification of temporary problems. Therefore, it is necessary to explore together with the owner of the claim database whether more recent data, for example by monthly updates, could possibly be made available to the Inspectorate.

###  Implications for Research and Practice

 Although the change of identifying practices with problems using these existing data increased moderately, it is still better than collecting new data for supervision for the Inspectorate since there is no extra administrative burden for healthcare professionals. Especially GP practices are often small with limited opportunities for administrative recording. In the Netherlands, there is therefore an intense debate about collecting data for the purposes of supervision. It is important to restrict the administrative burden in order to achieve acceptance by the healthcare professionals and their associations. The Dutch Health and Youth Care Inspectorate is continuously looking for alternatives for collecting additional indicators by healthcare professionals. However, we learned from our analysis that using an existing database is not sufficient to identify substandard care. Therefore, collecting data especially for supervision purposes might be inevitable.

 As mentioned in the introduction the Inspectorate uses existing public data from patient rating sites to identify healthcare providers with problems.^9,10^ A combination of these indicators from a patient perspective with our indicators might increase the predictive value.

 The Dutch Health and Youth Care Inspectorate is considering using the indicators for its daily supervision but has indicated that further research is needed. The 3 identified themes and indicators might also be used by healthcare inspectorates in other countries. Extremes in costs, prescribing, emergency admission to a hospital during the weekend and an extremely high workload are internationally acknowledged threads for patient safety in primary care. Using claim data for supervision might also be an interesting option for other countries to identify patient safety risks. However, this will strongly depend on the existence of a claim database, its completeness and accuracy and permission to use the data. Alternatives are other administrative databases such as Medicare data in the United States or clinical audit data.

 The access to the claim database also offers the Inspectorate other opportunities. The database might be used for datamining in order to identify trends and patterns that might indicate safety risks. A major advantage of using methods such as machine learning techniques is that more variables can be explored which may predict substandard care. However, the quality of the data is often a concern in healthcare.^17^ Future research has to identity the opportunities offered by this health claim database for the purposes of datamining.

## Conclusion

 The Dutch Health and Youth Care Inspectorate might use claim data to calculate indicators on costs, the prescribing of reserve antibiotics and emergency admissions during the weekend, when setting priorities for its visits to GP practices. However, we had not sufficient cases of GP practices with safety problems in our database to assess, reliably, the sensitivity and specificity of the combination of indicators. Therefore, visits by the Health and Youth Care Inspectorate to GP practices in order to identify substandard care are necessary to validate using these indicators.

## Acknowledgements

 We would like to thank VEKTIS for making the data available and Bas Kluitenberg of the Dutch Health and Youth Care Inspectorate of helping with the analyses.

## Ethical issues

 We used administrative data for analysis with the permission of the supervising body VEKTIS. For the preparing interviews with experts, according to Dutch law, no approval is necessary from an ethics committee.

## Competing interests

 Authors declare that they have no competing interests.

## Authors’ contributions

 RBK and IB designed the study. RBK wrote a first draft of the manuscript, RA, CB and SR analyzed and, together with RBK and IB, interpreted the data. All authors interpreted the analyses, commented on the first draft and read and approved the final manuscript.

## Funding

 This study was funded by ZonMw that had no role in the design of the study and collection, analysis, and interpretation of data and in writing the manuscript.

## Supplementary files


Supplementary file 1. Topic List.
