# Supplementary material for: Can the Use of Health Insurance Claim Data Benefit the Risk-Based Supervision of General Practitioner Practices? An Exploratory Study in the Netherlands
Source: Int J Health Policy Manag. 2020 Dec 19;11(7):1009–16. doi: 10.34172/ijhpm.2020.242 (PMC9808193; doi:10.34172/ijhpm.2020.242)
Supplement: Supplementary file 1 — Topic List. [file ijhpm-11-1009-s001.pdf]

Supplementary file 1. Topic List

Professional organizations

Personal characteristics

- What is your age, gender, (job) position, work experience?

Risk-based supervision

- When does care not comply to basic quality standards?
    - Optional: explain the meaning of irresponsible care and danger to patient safety
    - Name a few specific situations
- What are the most important risk factors that can lead to substandard quality of care?
    - Optional: which missing preconditions/external influences can potentially lead to substandard quality of care?
- Which healthcare professionals are most likely to be affected by these risk factors?
    - What are the characteristics of these practices or healthcare professionals?
- How can these risk factors be made visible?
    - (External) visitations
    - Complaints
    - Notifications
    - Patient experiences
    - Refresher courses and further training
    - Quality indicators
- Which role does the [professional organization] play in terms of monitoring these risk factors?
    - Active versus passive role
    - Preventive or reactive
- To which extend does the [professional organization] support the [care professionals] in order to prevent these risks?
    - Refresher courses and further training [accreditation]
    - Registration/contact point
    - Confidant
- In what way can the [professional organization] collaborate with the Health and Youth Care Inspectorate to monitor and prevent these risks?

Health and Youth care Inspectorate

Personal characteristics

- What is your age, gender, (job) position, work experience?

Risk-based supervision

- When does care not comply to basic quality standards?
    - Optional: explain the meaning of irresponsible care and danger to patient safety
    - Name a few specific situations
- What are the most important risk factors that can lead to substandard quality of care?
    - Optional: which missing preconditions/external influences can potentially lead to substandard quality of care?
- Which healthcare professionals are most likely to be affected by these risk factors?
    - What are the characteristics of these practices or care professionals?
- Which role can risk-based supervision play in the tracking down risks related to substandard quality of care?
    - Which sources of information can be useful for risk-based supervision?
    - In what way would these sources be useful?
- [Based on previous interviews] we received information that professional organizations use the following sources of information [.....]
    - In your opinion, to what extend are these sources useful for risk-based supervision?
- What is your perspective on the insights we have gained on risk-based supervision or risk factors from the literature and professional organizations?
    - Optional: elaborate on the literature
- Which role can the Healthcare Inspectorate play concerning the prevention of these risks (in collaboration with the professional organization)?

Literature on which topic list was based

Risk-based supervision in healthcare

• Adil M. Risk-based regulatory system and its effective use in health and social care. J R Soc Promot Health. 2008 Jul;128(4):196-201. PubMed PMID: 18678116. https://www.ncbi.nlm.nih.gov/pubmed/18678116

• Bardsley M, Spiegelhalter DJ, Blunt I, Chitnis X, Roberts A, Bharania S. Using routine intelligence to target inspection of healthcare providers in England. Qual Saf Health Care. 2009 Jun;18(3):189-94. doi: 10.1136/qshc.2007.024802. PubMed PMID: 19468000. https://www.ncbi.nlm.nih.gov/pubmed/19468000

• Beaussier AL, Demeritt D, Griffiths A, Rothstein H. Accounting for failure: risk-based regulation and the problems of ensuring healthcare quality in the NHS. Health Risk Soc. 2016 May 18;18(34):205-224. PubMed PMID: 27499677; PubMed Central PMCID: PMC4950452. https://www.ncbi.nlm.nih.gov/pubmed/27499677

• Denham L. Phipps , Peter R. Noyce , Kieran Walshe , Dianne Parker & Darren M. Ashcroft (2011) Risk-based regulation of healthcare professionals: What are the implications for pharmacists?, Health, Risk & Society, 13:3, 277-292, DOI: 10.1080/13698575.2011.558624 http://www.tandfonline.com/doi/pdf/10.1080/13698575.2011.558624?needAccess=true

• Lloyd-Bostock, Sally and Hutter, Bridget M. (2008) Reforming regulation of the medical profession: the risks of risk-based approaches. Health, Risk and Society, 10 (1). pp. 69-83. ISSN 1369-8575. http://eprints.lse.ac.uk/5928/

• Asenova, D., Stein, B., & Marshall, A. (2011). An innovative approach to risk and quality assessment in the regulation of care services in Scotland. Journal of Risk Research, 14(7), 859879. 10.1080/13669877.2011.571780. http://researchonline.gcu.ac.uk/portal/en/publications/an-innovative-approach-to-risk-andquality-assessment-in-the-regulation-of-care-services-in-scotland(99dbd618-a86d-433c-92770db680d55bf5).html

• Griffiths A, Beaussier AL, Demeritt D, Rothstein H. Intelligent Monitoring? Assessing the ability of the Care Quality Commission's statistical surveillance tool to predict quality and prioritise NHS hospital inspections. BMJ Qual Saf. 2016 Apr 18. pii: bmjqs-2015-004687. doi: 10.1136/bmjqs-2015-004687. [Epub ahead of print] PubMed PMID: 27090161. http://qualitysafety.bmj.com/content/early/2016/04/15/bmjqs-2015-004687.abstract

• Ian Blunt, Xavier Chitnis, Adam Roberts. Automated Risk Detection - What are the Key Elements Needed to Create a Multi-source, Pattern-based Risk Detection System? 2009, Conference paper. https://www.researchgate.net/profile/Ian_Blunt/publication/221334533_Automated_Risk_Dete ction_-_What_are_the_Key_Elements_Needed_to_Create_a_Multi-source_Patternbased_Risk_Detection_System/links/00b4951c18e7d61118000000.pdf

• Spiegelhalter, D., Sherlaw-Johnson, C., Bardsley, M., Blunt, I., Wood, C. and Grigg, O. (2012), Statistical methods for healthcare regulation: rating, screening and surveillance. Journal of the Royal Statistical Society: Series A (Statistics in Society), 175: 1–47. doi:10.1111/j.1467985X.2011.01010.x. http://onlinelibrary.wiley.com/doi/10.1111/j.1467-985X.2011.01010.x/full

Risk-based supervision in general

• Black, J. and Baldwin, R. (2012), When risk-based regulation aims low: A strategic framework. Regulation & Governance, 6: 131–148. doi:10.1111/j.1748-5991.2012.01127.x http://onlinelibrary.wiley.com/doi/10.1111/j.1748-5991.2012.01127.x/abstract

• Black, J., & Baldwin, R. (2012). When risk-based regulation aims low: approaches and challenges. Regulation & Governance, 6, 2-22. http://onlinelibrary.wiley.com/doi/10.1111/j.1748-5991.2011.01124.x/abstract

• Black, J., & Baldwin, R. (2010). Really responsive risk-based regulation. Law & Policy, 32, 181213. http://onlinelibrary.wiley.com/doi/10.1111/j.1467-9930.2010.00318.x/full

• Prof. mr. drs. F.C.M.A. Michiels en Dr. ir. H. Paul, 'Risicogestuurd toezicht en systeemtoezicht', TvT 2010-3, p. 74-77 http://www.bjutijdschriften.nl/tijdschrift/tijdschrifttoezicht/2010/3/TvT_18798705_2010_001_003_006.pdf

• Mr. dr. Ir. Bart Custers, 'Risicogericht toezicht, profiling en Big Data', TvT 2014-3, p. http://www.bjutijdschriften.nl/tijdschrift/tijdschrifttoezicht/2014/3/TvT_18798705_2014_005_003_002

• Rothstein H, Irving P, Walden T, Yearsley R. The risks of risk-based regulation: insights from the environmental policy domain. Environ Int. 2006 Dec;32(8):1056-65. Review. PubMed PMID: 16842849. https://www.ncbi.nlm.nih.gov/pubmed/16842849

• Mr. dr. Paul Verbruggen en Dr. ir. Tetty Havinga, 'Metatoezicht op voedselveiligheid', TvT 20141, p. 6-32 http://www.bjutijdschriften.nl/tijdschrift/tijdschrifttoezicht/2014/1/TvT_18798705_2014_005_001_002

Supervision in healthcare

• Dr. Annemiek Stoopendaal, Dr. ing. Martin de Bree, Drs. Franske Keuter e.a. , 'Systeemtoezicht in de Nederlandse gezondheidszorg. Een experimentele innovatie van toezicht.', TvT 2014-2, p. 27-46 http://www.bjutijdschriften.nl/tijdschrift/tijdschrifttoezicht/2014/2/TvT_18798705_2014_005_002_003

• Dr. M.E. Honingh en Dr J.K. Helderman, 'Voor wie of wat is systeemtoezicht zinvol?', TvT 2010-2, p. 6-25 http://www.bjutijdschriften.nl/tijdschrift/tijdschrifttoezicht/2010/2/TvT_18798705_2010_001_002_002

• Verver D, Merten H, Robben P, Wagner C. Supervision of care networks for frail community dwelling adults aged 75 years and older: protocol of a mixed methods study. BMJ Open. 2015 Aug 25;5(8):e008632. doi: 10.1136/bmjopen-2015-008632. PubMed PMID: 26307619; PubMed Central PMCID: PMC4550721. https://www.ncbi.nlm.nih.gov/pubmed/26307619
• Dodds A. Kodate N. Accountability, organisational learning and risks to patient safety in England: Conflict or compromise? Health, Risk and Society. Pages 327-346, 27 may 2011. http://www.tandfonline.com/doi/abs/10.1080/13698575.2011.575454
• Carney T, Beaupert F, Chiarella M, Bennett B, Walton M, Kelly PJ, Satchell CS. Health complaints and regulatory reform: Implications for vulnerable populations? J Law Med. 2016 Mar;23(3):650-61. Review. PubMed PMID: 27323641. https://www.ncbi.nlm.nih.gov/pubmed/27323641

Indicators and supervision
• Dr. S. Adamini, Prof. dr. M. Canoy en Dr. W. Oortwijn, 'Kwaliteitsindicatoren in de zorg: hoe om te gaan met gaming en erosie van intrinsieke motivatie?', TvT 2011-1, p. 23-32 http://www.bjutijdschriften.nl/tijdschrift/tijdschrifttoezicht/2011/1/TvT_18798705_2011_002_001_003
• Mears A, Vesseur J, Hamblin R, Long P, Den Ouden L. Classifying indicators of quality: a collaboration between Dutch and English regulators. Int J Qual Health Care. 2011 Dec;23(6):637-44. doi: 10.1093/intqhc/mzr055. PubMed PMID: 21846732. http://intqhc.oxfordjournals.org/content/23/6/637.short
